# Lactate Transport via Glial MCT1 and Neuronal MCT2 Is Not Required for Synchronized Synaptic Transmission in Hippocampal Slices Supplied With Glucose

**DOI:** 10.1111/jnc.70251

**Published:** 2025-10-06

**Authors:** Lennart Söder, Felipe Baeza‐Lehnert, Babak Khodaie, Amr Elgez, Lena Noack, Andrea Lewen, Stefan Hallermann, Gernot Poschet, Karin Borges, Oliver Kann

**Affiliations:** ^1^ Institute of Physiology and Pathophysiology Heidelberg University Heidelberg Germany; ^2^ Carl‐Ludwig‐Institute of Physiology, Faculty of Medicine Leipzig University Leipzig Germany; ^3^ Metabolomics Core Technology Platform, Centre for Organismal Studies Heidelberg University Heidelberg Germany; ^4^ School of Biomedical Sciences, Faculty of Health, Medicine and Behavioural Sciences The University of Queensland Brisbane Queensland Australia; ^5^ Interdisciplinary Center for Neurosciences (IZN) Heidelberg University Heidelberg Germany

**Keywords:** aerobic glycolysis, brain energy metabolism, lactate oxidation, monocarboxylate transporter, neurotransmission, tissue oxygenation

## Abstract

The metabolite lactate (L‐lactate) has been hypothesized to represent an important energy source during brain activation. The contribution of lactate in fueling synchronized synaptic transmission during fast neural network oscillations underlying complex cortex function such as visual perception, memory formation, and motor activity is less clear, however. We explored the role of cellular lactate production and lactate transport (uptake and release) via the monocarboxylate transporters 1 and 2 (glial MCT1 and neuronal MCT2) during persistent gamma oscillations (frequency at around 40 Hz) and recurrent rhythmic events called sharp wave‐ripples (with “ripples” at around 250 Hz) in cultured rat and acute mouse hippocampal slices (ex vivo) that received energy substrate supply with glucose (D‐glucose) only. In addition, we assessed neuronal lactate dynamics during spontaneous activity (“resting state”) and during electrical stimulation (10 Hz) in mouse primary neuron‐astrocyte cultures (in vitro) receiving glucose only. We combined electrophysiology (local field potential recordings), tissue lactate analysis [ultra‐performance liquid chromatography‐mass spectrometry (UPLC‐MS)], and live‐cell fluorescence imaging [Förster resonance energy transfer (FRET) sensor Laconic]. We report that (1) lactate is produced during gamma oscillations when glucose is supplied and oxygen availability is unlimited (high oxygenation) for mitochondrial respiration. (2) The properties of gamma oscillations remain regular in the presence of the MCT1/2 blocker AR‐C155858. (3) By contrast, MCT1/2 blockade fully suppresses gamma oscillations when mainly lactate is supplied. (4) The properties of sharp wave‐ripples remain regular during MCT1/2 inhibition. (5) Lactate is produced in primary hippocampal neurons during spontaneous activity and electric stimulus‐induced excitation, and it accumulates in the neuronal cytosol during MCT1/2 inhibition. In conclusion, lactate is produced in cortical tissue, including neurons fueled by glucose only. Moreover, lactate transport and lactate exchange (“shuttling”) via glial MCT1 and neuronal MCT2 are not required to sustain synchronized synaptic transmission during fast neural network oscillations.

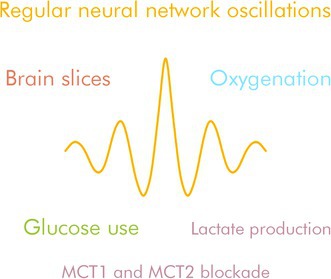

## Graphical abstract

Lactate has been hypothesized to represent an important energy source during brain activation. Here, we explored both lactate production and lactate transport across cell membranes in hippocampal slices that expressed natural neurotransmission reflected by fast neural network oscillations. Our data suggest that lactate is produced in hippocampal tissue, including neurons fueled by glucose only. Moreover, lactate transport and lactate “shuttling” via the glial monocarboxylate transporter 1 (MCT1) and the neuronal MCT2 are not required for synchronized synaptic transmission.

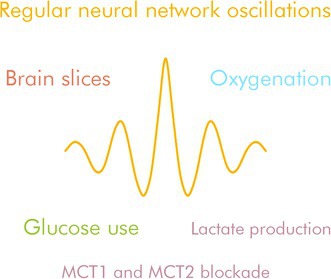

**Abbreviations**
ATP: adenosine‐5′‐triphosphate
CA: cornu ammonis
DAB: 1,4‐dideoxy‐1,4‐imino‐D‐arabinitol
fMRS: functional magnetic resonance spectroscopy
FRET: Förster resonance energy transfer
FWHM: full width at half‐maximum
GABA: γ‐aminobutyric acid
GLUT: glucose transporter
LDH: lactate dehydrogenase
LFP: local field potential
MCT: monocarboxylate transporter
Pi: inorganic phosphate
pO_2_
: interstitial partial pressure of oxygen
RRID: research resource identifier
UPLC‐MS: ultra‐performance liquid chromatography‐mass spectrometry

## 1 Introduction

Neuronal activation associates with a high energy demand and requires sufficient supply of glucose (D‐glucose) and oxygen for the synthesis of adenosine‐5′‐triphosphate (ATP) in glycolysis and oxidative metabolism in mitochondria (Dienel 2019a; Kann and Kovács 2007; Yellen 2018). ATP is essential to fuel ion pumps in neuronal membranes for maintaining ion concentration gradients that are finally used to establish neuronal excitability and synaptic transmission (Howarth et al. 2012; Kann 2024; Rae et al. 2024; Yellen 2018).

Glucose uptake by neurons mainly occurs through the glucose transporters GLUT3 and also GLUT4 (Ashrafi et al. 2017; Koepsell 2020; Simpson et al. 2007). In addition, plasma membrane monocarboxylate transporters (MCTs) permit the usage of supplemental fuels such as lactate, pyruvate, and ketone bodies (Achanta and Rae 2017; Barros et al. 2021; McKenna et al. 2012). Neurons predominantly express the MCT2 subtype, whereas MCT1 is mainly present in astrocytes and oligodendrocytes (Medel et al. 2022; Pierre et al. 2000; Rafiki et al. 2003; Simpson et al. 2007). Lactate transport in neurons and glial cells via MCTs mainly depends on the local concentration gradients of lactate and protons (DiNuzzo et al. 2024; Hertz et al. 2014; Mächler et al. 2016).

The metabolite lactate (L‐lactate) can be produced and released by various cell types of the body, such as skeletal muscle, immune, and brain cells (Brooks 2018; Kann 2024). For lactate production, pyruvate—the end product of glucose in glycolysis—is reduced by the bidirectional redox enzyme lactate dehydrogenase (LDH), which requires nicotinamide adenine dinucleotide (Dienel 2019a; Rae et al. 2024). In the brain, lactate production can occur in the presence and absence of sufficient oxygen levels, referred to as aerobic and anaerobic glycolysis, respectively (Barros et al. 2021; Dienel 2019a; Fox et al. 1988; Horvat et al. 2021; Madsen et al. 1999; McIlwain and Tresize 1956). The fractions of aerobic and anaerobic glycolysis in vivo are difficult to assess, however. One reason is that microregional cortical hypoxia may occur (Beinlich et al. 2024; Devor et al. 2011; Kasischke et al. 2011; Schneider et al. 2019).

Lactate can serve as a supplemental neuronal fuel (Cox and Bachelard 1988; Hollnagel et al. 2020; Quistorff et al. 2008; Rasmussen et al. 2010), and an astrocyte‐to‐neuron lactate shuttle hypothesis has been debated (Brooks 2018; Dienel 2017, 2019a; Kann 2024; Magistretti and Allaman 2018; Pellerin and Magistretti 2012; Suzuki et al. 2011; Yellen 2018). For ATP synthesis, lactate is linked to oxidative metabolism in mitochondria. This requires conversion of lactate to pyruvate through LDH, the tricarboxylic acid cycle (Szent‐Györgyi‐Krebs cycle), and molecular oxygen as the final electron acceptor at the respiratory chain (Brooks 2018; Dienel 2019a). Therefore, lactate oxidation in neurons and glial cells associates with oxygen consumption (Chausse et al. 2024; Hollnagel et al. 2020; Kann 2024).

In the present study, we explored the role of lactate production and lactate transport via glial MCT1 and neuronal MCT2 during synchronized synaptic transmission underlying fast neural network oscillations.

We primarily used cultured hippocampal slices (slice cultures) that exhibited fast neural network oscillations in the gamma‐frequency band (30–70 Hz) (Kann et al. 2011; Schneider et al. 2015). In addition, we used acute hippocampal slices that exhibited recurrent sharp wave‐ripple activity (with “ripples” frequency at around 250 Hz) (Schlingloff et al. 2014; Schneider et al. 2019). Hippocampal gamma oscillations and sharp wave‐ripples require precisely timed (synchronized) mutual synaptic transmission between glutamatergic pyramidal cells and γ‐aminobutyric acid (GABA)ergic interneurons (Gulyás et al. 2010; Schieferstein et al. 2024; Traub et al. 1996). Both network oscillations underlie higher brain functions in vivo such as visual perception, motor activity, and memory formation (Cheyne et al. 2008; Colgin 2016; Gray et al. 1989; van Vugt et al. 2010). Gamma oscillations feature higher energy demands compared with sharp wave‐ripples and asynchronous network activity (Hollnagel et al. 2020; Kann 2016; Schneider et al. 2019).

For interfering with plasma membrane lactate transport, we used the cell‐permeable compound AR‐C155858. This blocker is highly specific to MCT1 and MCT2 (Ovens, Davies, et al. 2010; Ovens, Manoharan, et al. 2010). It has been used in the low micromolar concentration range to effectively inhibit lactate transport (i.e., lactate uptake and release) in neurons and astrocytes in various experimental models (Baeza‐Lehnert et al. 2019; Denker and Dringen 2024; Díaz‐García et al. 2017; Köhler et al. 2018; Sotelo‐Hitschfeld et al. 2015; Wu et al. 2024) as well as in adenocarcinoma cell lines and rat erythrocytes (Blaszczak et al. 2022; Ovens, Davies, et al. 2010).

We report that lactate is produced in cortical tissue, including neurons fueled by glucose only, and that lactate transport via MCT1 and MCT2 is not required for synchronized synaptic transmission in hippocampal slices.

## 2 Materials and Methods

### 2.1 Rats and Mice

For preparations of cultured hippocampal slices (organotypic hippocampal slice cultures), male Wistar rat pups (Janvier Laboratories, Le Genest‐Saint‐Isle, France, cat. no. RN‐WI‐F) were used. For acute hippocampal slices, male C57BL/6 mice (Charles River Laboratories, Sulzfeld, Germany, RRID:IMSR_CRL:027) were used. Animals were kept under specific pathogen‐free conditions in conventional housing (mice: type II cages; rats: type IV cages) with standard bedding (ABBEDD LT‐E‐001). Housing density followed the recommendations of the German Society of Laboratory Animal Science. A 12:12 h light/dark cycle, ambient temperature of 22°C ± 2°C, and relative humidity between 50% and 60% were maintained during housing. Food (Altromin Rod 16 or Rod 18) and water were provided ad libitum. Environmental enrichment was provided in the form of cellulose‐based nesting materials (e.g., Nestlets) for mice and polycarbonate shelters for rats.

For primary hippocampal neuron cultures, male and female C57BL/6 mice (locally bred in the Animal Facility of the Faculty of Medicine, University of Leipzig, Germany) were used. Mice were housed in individually ventilated cages (Tecniplast GM500 Green Line Type II) in groups of 3 to 5 littermates under controlled conditions (temperature: 22°C ± 0.5°C, humidity: 55%) and maintained under a 12:12 h light/dark cycle with a 30 min dawn phase (lights on at 6 AM). Food and water were available ad libitum.

Experiments were conducted and reported in accordance with the ARRIVE guidelines. Handling and experiments were in accordance with the European directive 2010/63/EU and approved by the animal welfare office at Heidelberg University (licenses T‐37/21 and T‐27/24) and Leipzig University (license T‐01/21). In total, 36 rats and 16 mice were used.

### 2.2 Cultured Slices, Acute Slices and Primary Neuron‐Astrocyte Cultures

#### 2.2.1 Cultured Hippocampal Slices

Organotypic hippocampal slice cultures were prepared following previously described protocols (Lewen et al. 2020; Papageorgiou et al. 2016). Postnatal day 9 old male Wistar rats were killed by decapitation. Hippocampi were quickly removed, cooled to about 4°C in minimal essential medium saturated with 95% O_2_ plus 5% CO_2_ (vol/vol), and then cut into slices of 400 μm thickness using a McIlwain tissue chopper (Mickle Laboratory Engineering Company Ltd., Guildford, UK) under sterile conditions. Three slices with preserved hippocampal structures were placed on Biopore membrane inserts (Millicell standing inserts, Merck Millipore, Darmstadt, Germany, cat. no. PICM03050).

Cultured slices were maintained at 36.5°C in a Heracell incubator (Thermo‐Fisher Scientific, Dreieich, Germany) at the interface between the culture medium and humidified normal (room) air, enriched with 5% vol/vol CO_2_. The culture medium consisted of 50% minimal essential medium (Life Technologies, Darmstadt, Germany, cat. no. 11012044), 25% Hank's balanced salt solution (Sigma‐Aldrich, Taufkirchen, Germany, cat. no. H2387), 25% heat‐inactivated horse serum (Life Technologies, cat. no. 26050088), and an additional 2 mM L‐glutamine (Life Technologies, cat. no. 25030024), and it was titrated to pH 7.3 with Trisbase. The glucose concentration in the culture medium was approximately 4 mM, and the partial oxygen pressure (pO_2_) in the slice core was near the physiological range (Huchzermeyer et al. 2013; Schneider et al. 2015). Such glucose levels were shown to promote mitochondria content and oxidative phosphorylation in neurons in vitro (Swain et al. 2025). The culture medium was free of antibiotics. The culture medium (1 mL) was exchanged three times per week. Experiments were performed at days in vitro 12 to 16.

#### 2.2.2 Acute Hippocampal Slices

Acute hippocampal slices were prepared following previously described protocols (Kann et al. 2011; Khodaie et al. 2024). C57BL/6 mice at the age of 4–6 weeks were killed by decapitation during anesthesia with general inhalation anesthetics (1.5% vol/vol isoflurane in a gas mixture of 70% nitrous oxide and 30% O_2_; Isofluran Baxter, Baxter Deutschland GmbH, Unterschleißheim, Germany, cat. no. HDG9623). The brain was quickly removed and maintained in ice‐cold (3°C) recording solution (see below), saturated with 95% O_2_ plus 5% CO_2_. After removal of the frontal brain structures and the cerebellum, horizontal entorhinal‐hippocampal slices of 400 μm thickness were cut using a vibratome (VT 1200S, Leica Microsystems, Nussloch, Germany). These “acute” slices were stored in a custom‐built interface chamber at 34°C ± 1°C for recovery of at least 2 h before the start of electrophysiological recordings (Kann et al. 2011; Schneider et al. 2019). Isoflurane and nitrous oxide (laughing gas) are volatile anesthetics that rapidly flow off the tissue in the interface recording chamber (95% O_2_ plus 5% CO_2_). Therefore, anesthesia did not interfere with neurotransmission and energy metabolism in slice experiments. Slices from the ventral and the adjacent intermediate hippocampus were used for experiments and randomly assigned to experimental groups.

#### 2.2.3 Primary Hippocampal Neuron‐Astrocyte Cultures

Hippocampal neurons and astrocytes were prepared from C57BL/6 mice. Postnatal days 0 to 2 old mice were killed by decapitation. The brain was quickly removed, and the hippocampi were dissected free of meninges. For each preparation, six hippocampi from three pups of the same litter were used. In total, four similar preparations were conducted. The tissue was pooled and enzymatically dissociated in Hank's balanced salt solution (Gibco, Grand Island, NY, USA, cat. no. 14175095) containing 0.25% trypsin–EDTA (Thermo‐Fisher Scientific, cat. no. 25200056) for 15 min at 37°C, and then the digestion was stopped by the addition of Neurobasal medium (Gibco, Grand Island, NY, USA, cat. no. A2477501) containing 10 mM glucose, 2% B‐27 supplement (Gibco, cat. no. 17504044), 1% Glutamax (Gibco, cat. no. 35050061), and 5% newborn calf serum (Sigma‐Aldrich, cat. no. N4762). After mechanical dissociation, the cells were plated in poly‐L‐lysine‐coated 25 mm glass coverslips for 3 h, followed by the removal of medium and the addition of 2 mL of serum‐free Neurobasal Medium (Gibco) containing 10 mM glucose, 2% B‐27 supplement (Gibco), 1% Glutamax (Gibco), and 10 units/mL penicillin/10 μg/mL streptomycin (Thermo‐Fisher Scientific, cat. no. 15140122). The primary neuron‐astrocyte cultures were kept at 37°C in humidified normal (room) air plus 5% CO_2_ in a Forma Steri‐cycle i160 incubator (Thermo‐Fisher Scientific, Langensebold, Germany), and 2/3 of the medium was replaced every 3 days. Cells were infected at day in vitro 7 with an AAV/DJ‐hsyn‐Laconic viral construct to express the genetically encoded lactate‐sensitive sensor Laconic in neurons (San Martín et al. 2013; RRID:Addgene44238). Experiments were performed at days in vitro 15–17.

### 2.3 Electrophysiological Recordings

#### 2.3.1 Interface Recording Chamber

Cultured slices on Biopore membrane inserts and acute slices were transferred to a custom‐built interface chamber for electrophysiological recordings (Huchzermeyer et al. 2013; Papageorgiou et al. 2016). In this recording chamber, cultured slices and acute slices were maintained at the interface between recording solution and the experimental gas mixture.

Intact Biopore membrane inserts ensure rapid and efficient supply of oxygen, energy substrates, and compounds through the recording solution (3.4 mL/min flow rate) that flows underneath. Acute slices were maintained on two layers of lens filter paper (The Tiffen Company, Hauppauge, NY, USA) and supplied through the recording solution (1.8 mL/min flow rate). The interface chamber permits constant oxygen supply (rate 1.5 L/min) with the humidified experimental gas mixture (95% O_2_ plus 5% CO_2_). Energy substrates and compounds were applied via the recording solution for > 30 min, which is sufficient to achieve tissue saturation (Galow et al. 2014; Hollnagel et al. 2020).

#### 2.3.2 Recording Solutions and Compounds

For preparation and recovery of acute slices, the standard solution (artificial cerebrospinal fluid) contained 129 mM NaCl, 3 mM KCl, 1.25 mM NaH_2_PO_4_, 1.8 mM MgSO_4_, 1.6 mM CaCl_2_, 21 mM NaHCO_3_, and 10 mM D‐glucose (Kann et al. 2011; Schneider et al. 2015). For electrophysiological experiments in acute and cultured slices, the recording solution was similar but with 5 mM glucose. In some slice culture experiments, 2.5 mM glucose or 1 mM glucose plus 8 mM lactate were used. Standard and recording solutions were saturated with 95% O_2_ plus 5% CO_2_, resulting in a pH of 7.4. Electrophysiological recordings were performed at 33°C ± 1°C (cultured slices) and 34°C ± 1°C (acute slices).

Gamma oscillations were induced by continuous application of the cholinergic receptor agonist acetylcholine (Sigma‐Aldrich, cat. no. A6625) and the acetylcholine‐esterase inhibitor physostigmine (Tocris Bioscience, Bristol, UK via BioTechne, Wiesbaden‐Nordenstadt, Germany, cat. no. 0622) in rat cultured slices (Kann et al. 2011; Huchzermeyer et al. 2013). This pharmacological approach mimics cholinergic input to the hippocampus in vivo (Fischer et al. 2014; Kann et al. 2011). Sharp wave‐ripples occur spontaneously in mouse acute slices in common recording solutions (Schlingloff et al. 2014; Schneider et al. 2019).

To inhibit glycogen phosphorylase activity, 1,4‐dideoxy‐1,4‐imino‐D‐arabinitol (DAB) was used (Galow et al. 2014; Walls et al. 2008). Cultured slices were preincubated with DAB (1 mM) for 60 min in the incubator, and they further received DAB (300 μM) via the recording solution during electrophysiological recordings.

D‐glucose was purchased from Millipore (cat. no. 1.08342.1000), Na‐L‐Lactate from Thermo‐Fisher Scientific (cat. no. L14500.14), the MCT1/2 inhibitor AR‐C155858 from MedChemExpress (Monmouth Junction, NJ 08852, USA, via Biozol, Eching, Germany, cat. no. HY‐13248), and DAB from Cayman Chemical Company (Ann Arbor, Michigan, USA, via Biomol, Hamburg, Germany, cat. no. Cay20939).

#### 2.3.3 Local Field Potential Recordings

Gamma oscillations were recorded with glass electrodes (resistance of 1–2 MΩ) that were made from GB150F‐8P borosilicate capillaries (Science Products GmbH, Hofheim, Germany) using a horizontal micropipette puller (DMZ Zeitz‐Puller, Zeitz‐Instruments Vertriebs GmbH, Martinsried, Germany). The glass electrodes contained a silver‐chloride wire and were back‐filled with recording solution. Sharp wave‐ripples were recorded with carbon fiber electrodes (Kation Scientific, Minneapolis, MN, USA). The electrodes were positioned in stratum pyramidale of the cornu ammonis (CA) 3 region using a mechanical micromanipulator (MX‐4, Narishige International Ltd., London, UK).

The extracellular local field potential (LFP) was recorded with an EXT 10‐2F amplifier in an EPMS‐07 housing (npi Electronic GmbH, Tamm, Germany), low‐pass filtered at 3 kHz, and digitized at 10 kHz using a CED 1401 interface and Spike2 software (Cambridge Electronic Design, Cambridge, UK) (Hollnagel et al. 2020; Lewen et al. 2020; Papageorgiou et al. 2016).

### 2.4 FRET Imaging With Laconic

#### 2.4.1 Fluorescence Imaging in Primary Hippocampal Neurons

In the imaging setup, a coverslip with primary neuron‐astrocyte cultures was mounted in a RC‐21BRFS recording chamber (Warner Instruments, Holliston, MA, USA) and perfused with recording solution that contained 112 mM NaCl, 3 mM KCl, 1.25 mM CaCl_2_, 1.25 mM MgSO_4_, 2 mM glucose, 10 mM HEPES, and 24 mM NaHCO_3_. The recording solution was saturated with room air plus 5% CO_2_, resulting in a pH of 7.4.

Neurons were identified by Laconic expression, and fluorescence imaging was performed at 35°C–36°C using an Axio‐Observer Z1 microscope (Zeiss, Jena, Germany), a Plan‐Apochromat 20×/0.8 objective, and an Axiocam 506 camera (688 × 552 pixels, 4 × 4 binning, pixel size 0.91 × 0.91 μm), with acquisition times of 40 ms for the donor fluorophore (mTFP) and 60 ms for Förster resonance energy transfer (FRET). The two channels were recorded with the following filter sets: mTFP, excitation 436/25 nm, beam splitter 455 nm, and emission 480/40 nm; FRET, excitation 436/25 nm, beam splitter 455 nm, and emission 535/30 nm. Regions of interest, each containing a single neuronal cell body (soma), were defined manually using FIJI (Schindelin et al. 2012). Background‐subtracted mean fluorescence intensities averaged over all pixels within a region of interest were determined for the mTFP and FRET channels and subsequently used to calculate the mTFP/FRET ratio (Baeza‐Lehnert et al. 2020).

#### 2.4.2 Electrical Field Stimulation

Primary hippocampal neurons co‐cultured with astrocytes were field‐stimulated using a RC‐21BRFS chamber (Warner Instruments, Holliston, MA, USA) and a Grass s48 square pulse stimulator (Grass Instrument division, West Warwick City, RI, USA) connected to a SIU5 stimulus isolation unit (Grass Instrument division). Action potentials were evoked in neurons with 1 ms electrical stimuli creating field potentials of ~10 V/cm via platinum–iridium electrodes. Neurons were activated with trains of hundred electrical stimuli (10 s, 10 Hz).

### 2.5 Determination of Tissue Lactate

Biopore membrane inserts, which carried cultured slices exhibiting gamma oscillations in the presence of glucose (5 mM) or glucose (1 mM) plus lactate (8 mM) for > 30 min, were removed from the interface recording chamber and held at room air. Three cultured slices (= one sample) were immediately collected with an ice‐cold spoon, shock‐frozen in dry ice, and kept in a 1.5 mL tube at −80°C. Each sample was extracted in 0.15 mL ice‐cold methanol with sonication on ice. Determination of organic acids was performed according to previous reports via ultra‐performance liquid chromatography‐mass spectrometry (UPLC‐MS) after specific derivatization (Chausse et al. 2024; Uran et al. 2007).

### 2.6 Data Analysis and Statistics

Offline analysis of LFPs was performed in MATLAB 2023 (The MathWorks Inc., Natick, MA, USA) using custom‐made scripts (Hollnagel et al. 2020; Chausse et al. 2024).

For gamma oscillation analysis, data segments (300 s) were filtered using a low‐pass Butterworth algorithm at a 200 Hz corner frequency and processed with Welch's algorithm with a Hamming window size of 8192 points for the calculation of power spectral density, resulting in a bin size of 1.2207 Hz. The presence of gamma oscillations was defined when the oscillation frequency was ≥ 20 Hz at 33°C ± 1°C (corrected for the temperature in the interface recording chamber) (Schneider et al. 2015) and when the autocorrelation function permitted the computation of an exponential decay function in at least six out of ten 30 s segments within the data segments (300 s). Peak frequency (“frequency”), peak power (“power”), and full width at half‐maximum (FWHM) of gamma oscillations were calculated from entire data segments (300 s). Because gamma oscillations tend to change frequency over time in the interface recording chamber, the properties of gamma oscillations were analyzed in independent groups of cultured slices in the same time window (control conditions versus MCT1/2 blocker).

For sharp wave‐ripple analysis, data segments (300 s) were separated into slow (sharp wave) and fast (“ripples”) components by offline filtering. The slow component was obtained by low‐pass filtering (FFT filter, cut frequency 45 Hz) and used for event detection and calculation of amplitude. The ripple component was isolated by a band‐pass filter (FFT filter, pass‐band frequency 120–400 Hz) (Hollnagel et al. 2020).

Data originated from “*n*” slices (electrophysiology), samples (lactate determination), or neurons (fluorescence imaging) from “*N*” animals or coverslips (specified in figure legends). Individual data points (“*n*”) are presented by dots used for statistical analysis. Unless stated otherwise, box plots illustrate median and interquartile range, with whiskers indicating the minimum and maximum values. Columns represent mean ± standard error of the mean. Sample sizes were chosen based on previous studies (Baeza‐Lehnert et al. 2019; Hollnagel et al. 2020; Kann et al. 2011; Schneider et al. 2019). No test for outliers was conducted. For Laconic experiments, neurons presenting saturating FRET ratios prior to experimental interventions were excluded from the analysis.

Statistical significance (*p* < 0.05) was determined in GraphPad Prism 10 (GraphPad Software, California, USA) and jamovi Software (Version 2.6) retrieved from http://www.jamovi.org (The Jamovi Project 2025, Sydney, Australia). Data distribution was tested with the Shapiro–Wilk test. Statistical tests are specified in figure legends. Fisher's exact test was performed using absolute numbers, from which percentages were calculated. Details of each statistical test are provided in Table S1. Figures were created with GraphPad Prism 10 (GraphPad Software) and CorelDRAW (Corel Corporation, Ottawa, Ontario, Canada).

## 3 Results

### 3.1 Experimental Approach for Hippocampal Slice Preparations

**FIGURE 1:**
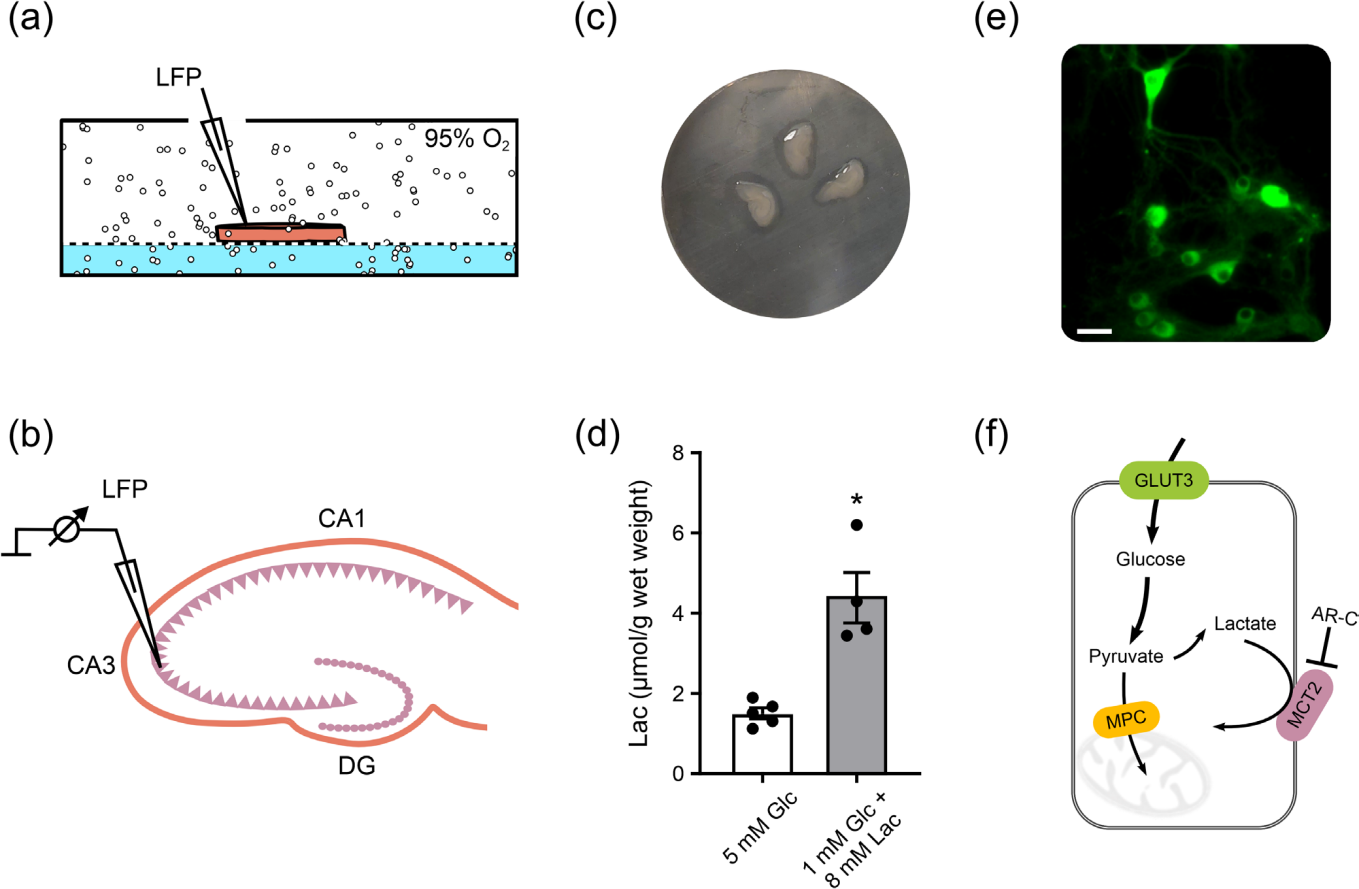
Experimental approach for investigating energy metabolism in hippocampal slices and primary hippocampal neurons. (a) Cultured slices and acute slices maintained on Biopore membrane inserts and lens filter paper, respectively, were examined in an interface recording chamber, with continuous exchange of gas mixture (95% O_2_ plus 5% CO_2_) and recording solution (light blue). (b) Local field potential (LFP) recordings were performed in stratum pyramidale of the CA3 region of the slices. CA1, cornu ammonis area 1; CA3, cornu ammonis area 3; DG, dentate gyrus. (c) Three cultured slices (organotypic hippocampal slice cultures) on Biopore membrane insert. (d) Lactate (Lac) content was determined by ultra‐performance liquid chromatography‐mass spectrometry (UPLC‐MS). Cultured slices exhibited gamma oscillations in the presence of glucose (Glc) or glucose plus lactate (Glc + Lac) for 60 min. For each sample (*n*), three cultured slices were shock‐frozen and pooled. *p = 0.0016, unpaired *t*‐test. For *n*/*N* (slices/animals): Glc, 15/5; Glc + Lac, 12/4. (e) Neurons expressing Laconic (Venus Channel) in primary hippocampal neuron‐astrocyte cultures (Figure 6). Bar corresponds to 20 μm. (f) Scheme about glucose metabolism (glycolysis and oxidative metabolism in mitochondria) and transport stop of endogenous lactate by the MCT1/2 blocker AR‐C155858 (AR‐C) in neurons supplied with glucose only. GLUT3, glucose transporter 3; MCT2, monocarboxylate transporter 2; MPC, mitochondrial pyruvate carrier.

We investigated gamma oscillations in cultured hippocampal slices of the rat in a custom‐built interface recording chamber (Figure 1a–c) that permits optimized delivery of oxygen and energy substrates, including fast application of compounds (Galow et al. 2014; Schneider et al. 2015).

The given oxygen fraction (%) and glucose concentrations (mM) refer to the slice surface (Figure 1a,d). They are significantly lower in the slice core (Section 4) (Huchzermeyer et al. 2013; Lourenço et al. 2019). Slice experiments were performed with glucose (5 mM) and 95% O_2_ (high oxygenation), with the aim of avoiding limitations in glycolysis and/or oxidative metabolism in mitochondria, especially in the slice core during gamma oscillations (Huchzermeyer et al. 2013; Kann 2024).

**FIGURE 2:**
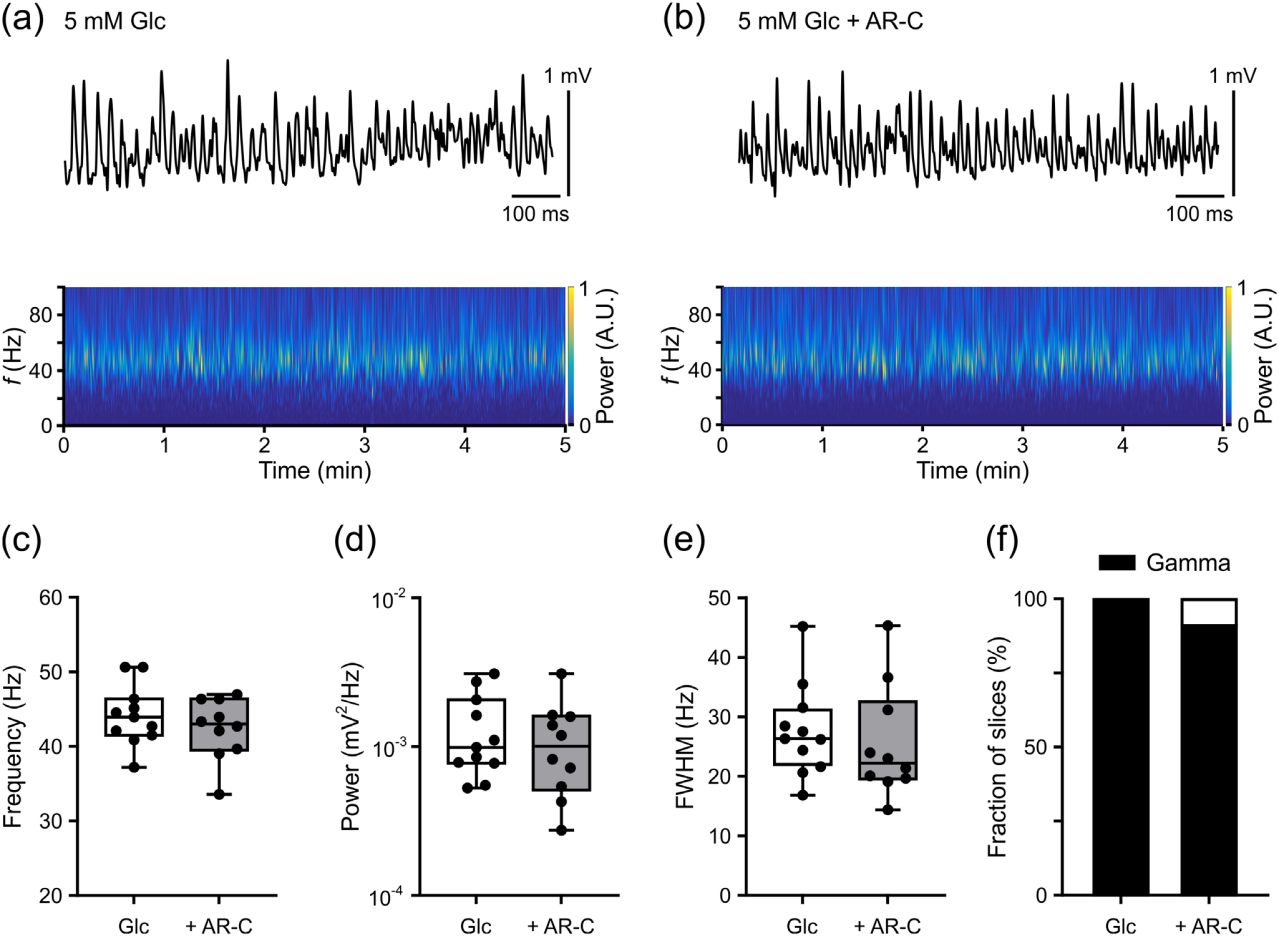
MCT1/2 inhibition during gamma oscillations in cultured slices supplied with glucose (5 mM). Gamma oscillations were induced by acetylcholine (2 μM) and physostigmine (1 μM). Local field potential (LFP) recordings were performed at 33°C ± 1°C. (a) and (b) Representative LFP traces (top) and Morlet wavelet transformations (bottom) from data segments (300 s) used for analysis (c–f). Slices received 5 mM glucose (Glc) in absence (a) and presence (b) of the MCT1/2 inhibitor AR‐C155858 (AR‐C) (2 μM). Heat‐scale colors encode for power in arbitrary units (A.U.). (c) Frequency, (d) power, and (e) full width at half‐maximum (FWHM) were analyzed in slices exhibiting gamma oscillations. Data were compared with an unpaired *t*‐test (c, e) and Mann Whitney's test (d) (no differences). (f) The presence of gamma oscillations was determined based on autocorrelation criteria in each slice (Section 2). Fractions were compared using Fisher's exact test (no difference). For *n*/*N* (slices/animals): Glc, 11/3; Glc + AR‐C, 11/3.

**FIGURE 3:**
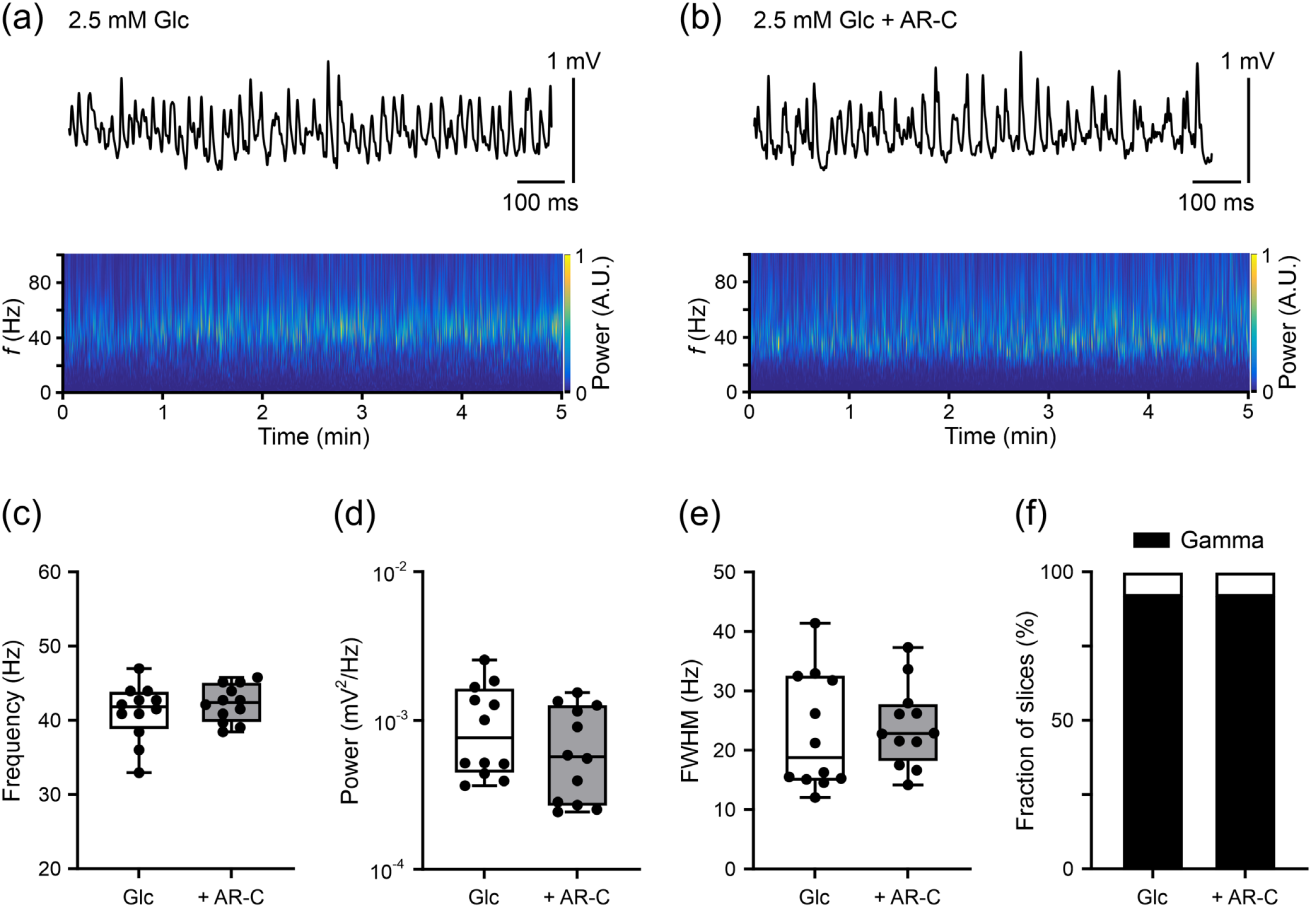
MCT1/2 inhibition during gamma oscillations in cultured slices supplied with lowered glucose (2.5 mM). Gamma oscillations were induced by acetylcholine (2 μM) and physostigmine (1 μM). Local field potential (LFP) recordings were performed at 33°C ± 1°C. (a) and (b) Representative LFP traces (top) and Morlet wavelet transformations (bottom) from data segments (300 s) used for analysis (c–f). Slices received 2.5 mM glucose (Glc) in absence (a) and presence (b) of the MCT1/2 inhibitor AR‐C155858 (AR‐C) (2 μM). Heat‐scale colors encode for power in arbitrary units (A.U.). (c) Frequency, (d) power and (e) full width at half‐maximum (FWHM) were analyzed in slices exhibiting gamma oscillations. Data were compared with unpaired *t*‐test (no differences). (f) The presence of gamma oscillations was determined based on autocorrelation criteria. Fractions were compared using Fisher's exact test (no difference). For *n*/*N* (slices/animals): Glc, 13/4; Glc + AR‐C, 13/4.

**FIGURE 4:**
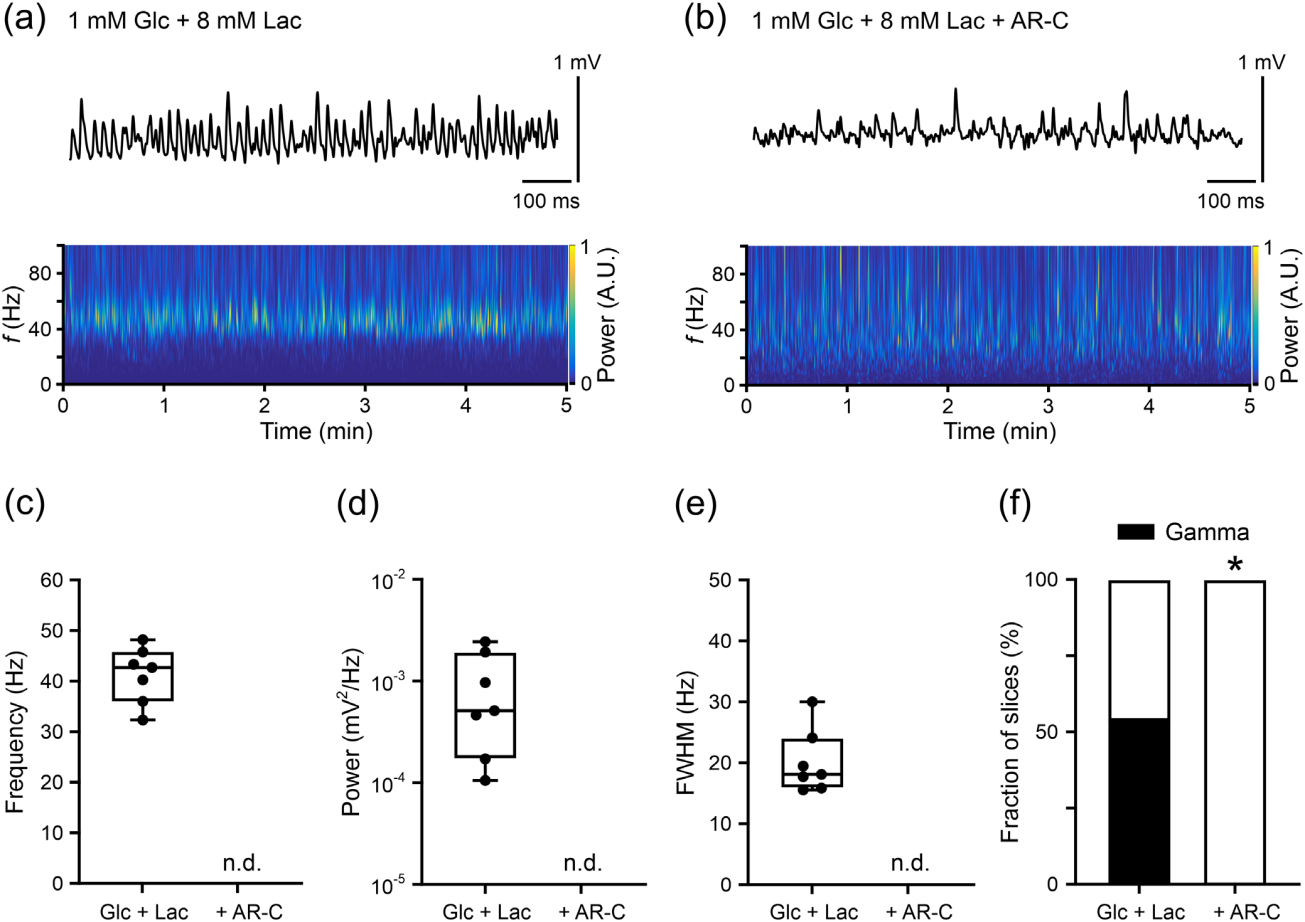
MCT1/2 inhibition during gamma oscillations in cultured slices supplied with low glucose (1 mM) plus high lactate (8 mM). Gamma oscillations were induced by acetylcholine (2 μM) and physostigmine (1 μM). Local field potential (LFP) recordings were performed at 33°C ± 1°C. (a) and (b) Representative LFP traces (top) and Morlet wavelet transformations (bottom) from data segments (300 s) used for analysis (c–f). Slices received 1 mM glucose (Glc) plus 8 mM lactate (Lac) in absence (a) and presence (b) of the MCT1/2 inhibitor AR‐C155858 (AR‐C) (2 μM). Heat‐scale colors encode for power in arbitrary units (A.U.). (c) Frequency, (d) power, and (e) full width at half‐maximum (FWHM) were analyzed in slices exhibiting gamma oscillations (n.d., not determined). (f) The presence of gamma oscillations was determined based on autocorrelation criteria. Fractions were compared using Fisher's exact test (*p = 0.006). Note that AR‐C fully suppressed gamma oscillations (b–f). For *n*/*N* (slices/animals): Glc + Lac, 13/5; Glc + Lac + AR‐C, 11/2.

Gamma oscillations (30 to 70 Hz) were induced by acetylcholine and physostigmine and recorded by an extracellular LFP electrode positioned in the hippocampal CA3 region (Figure 1b). Methodologically, changes in the LFP (mV) primarily arise from synaptic activity, that is, postsynaptic transmembrane currents (Einevoll et al. 2013; Gulyás et al. 2010; Schieferstein et al. 2024). In each experimental condition (control versus MCT1/2 blocker) (Figure 1f), the properties of gamma oscillations were analyzed in independent groups of cultured slices in the same time window (Figures 2, 3, 4).

**FIGURE 5:**
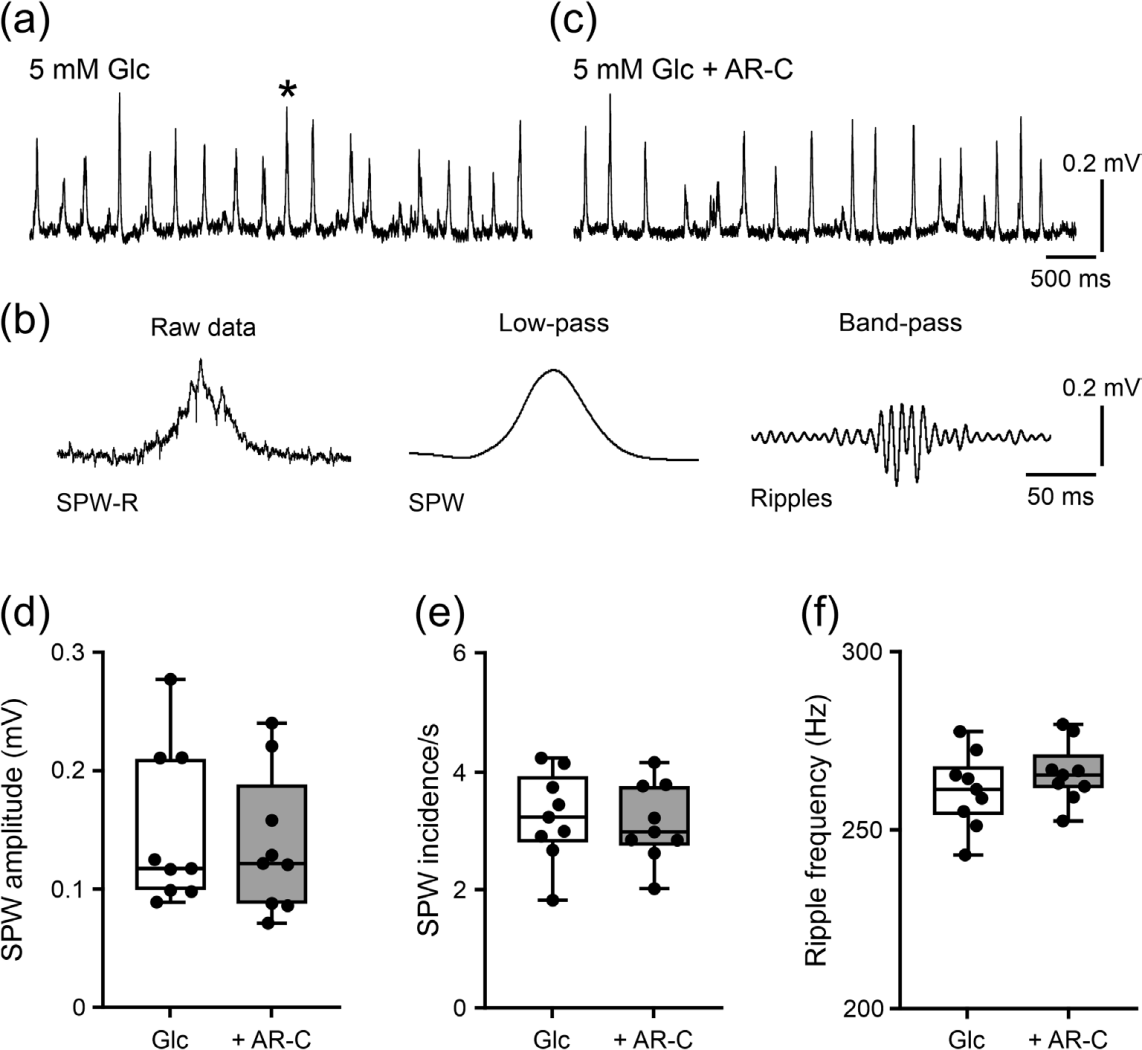
MCT1/2 inhibition during sharp wave–ripples in acute slices supplied with glucose (5 mM). Recurrent sharp wave–ripples (SPW‐R) occurred spontaneously in normal recording solution. Local field potential (LFP) recordings were performed at 34°C ± 1°C. (a) and (c) Representative LFP traces from data segments (300 s) used for analysis (d–f). Each slice received 5 mM glucose (Glc) in the absence (a) and, subsequently, in the presence (c) of the MCT1/2 inhibitor AR‐C155858 (AR‐C) (2 μM). (b) The sharp wave–ripples event marked in (a) with an asterisk is illustrated at higher temporal resolution (left). The slow sharp wave (SPW) component (middle) and the fast ripples component (right) were separated by low‐pass (< 45 Hz) and band‐pass (120–400 Hz) filtering, respectively. (d) Sharp wave amplitude, (e) sharp wave incidence, and (f) ripples frequency were analyzed for both conditions (a, c) in each slice. Data were compared with the Wilcoxon rank test (d) and paired *t*‐test (e, f) (no differences). Note that for each data point > 500 sharp wave–ripples were analyzed. For *n*/*N* (slices/animals): Glc, 9/4; Glc + AR‐C, 9/4.

We used a similar experimental approach for investigating sharp wave–ripples in acute hippocampal slices of the mouse (Figure 5).

### 3.2 MCT1/2 Inhibition During Gamma Oscillations in Cultured Slices

In the first series of experiments, we recorded gamma oscillations in the presence of glucose (5 mM) as the only energy substrate. In these control slices, persistent gamma oscillations were reliably present and showed frequencies at around 40 Hz (Figure 2a). From power spectral density computation (Section 2), we further quantified frequency, power, and FWHM of gamma oscillations in each slice (Figure 2c–e). Overall, the properties of these gamma oscillations were similar to those reported previously (Galow et al. 2014; Schneider et al. 2015; Vodovozov et al. 2018). Methodologically, it is thought that power generally increases with the number and synchrony of postsynaptic currents, whereas FWHM increases with jitter in their timing (Schneider et al. 2019).

After recordings of gamma oscillations, three slices per sample (*n* = 5) were shock‐frozen, and the tissue extracts were analyzed using UPLC‐MS (Section 2). We measured a lactate concentration of about 1.5 μmol/g wet weight in all slices supplied with glucose (5 mM) (Figure 1d). These data suggest lactate production by neurons and/or glial cells, even at high tissue oxygenation (not limiting lactate oxidation in mitochondria) and regular extracellular space in the tissue (Dienel 2019a; Jekabsons et al. 2017; Waagepetersen et al. 2000). However, we are technically unable to dissect “simple mass action” from “net production” of lactate. For comparison, we also analyzed tissue extracts from slices with attenuated gamma oscillations in the presence of low glucose (1 mM) plus high lactate (8 mM) (see below) (Figure 1d).

Next, we tested whether cellular lactate transport is required during gamma oscillations (Dienel 2019b; Newman et al. 2011; Suzuki et al. 2011). In another group of cultured slices, we therefore inhibited MCT1 and MCT2 with the specific high‐affinity blocker AR‐C155858 (Ovens, Davies, et al. 2010; Ovens, Manoharan, et al. 2010) in the concentration (2 μM) that had been successfully used in primary neurons and astrocytes, including hippocampal slices (Baeza‐Lehnert et al. 2019; Díaz‐García et al. 2017; San Martín et al. 2013). Opposite to α‐cyano‐4‐hydroxycinnamate (with the common abbreviation 4‐CIN), AR‐C155858 has not been reported to affect the mitochondrial pyruvate carrier (Angamo et al. 2016; Compan et al. 2015; Halestrap 1975; McKenna et al. 2001; Ovens, Davies, et al. 2010; Ovens, Manoharan, et al. 2010). Notably, application of AR‐C155858 did neither affect the properties of gamma oscillations (Figure 2b–e) nor the fraction of slices exhibiting gamma oscillations (Figure 2f).

In the second series of experiments, we recorded gamma oscillations in the presence of lower glucose (2.5 mM) only, with the aim to increase the cellular production of lactate by enhanced glycogenolysis, including putative lactate exchange (Cater et al. 2001; Swanson et al. 1992; Walls et al. 2009). Glycogen is mainly located in astrocytes—and to some extent in neurons—and its degradation provides net ATP yield as well as pyruvate that is converted to lactate or utilized in mitochondria (Dienel et al. 2025; Markussen et al. 2024; Oe et al. 2016). Slice cultures possess substantial glycogen stores that can be mobilized, for example, by different levels of glucose deprivation (Galow et al. 2014; Gramsbergen et al. 2003; Weis et al. 2021). With lower glucose (2.5 mM), persistent gamma oscillations were still reliably present and showed frequencies at around 40 Hz (Figure 3a). As a control experiment, we applied DAB—a potent inhibitor of glycogen phosphorylase activity (Section 2) (Galow et al. 2014; Walls et al. 2008). DAB disturbed gamma oscillations by decreasing the power and increasing the FWHM in the presence of glucose (2.5 mM) (Table S1), supporting the assumption that glycogenolysis and concomitant lactate production take place. Notably, application of AR‐C155858 in the presence of glucose (2.5 mM) did neither affect the properties of gamma oscillations (Figure 3b–e) nor the fraction of slices exhibiting gamma oscillations (Figure 3f), suggesting that glycogen‐derived lactate is not exchanged between astrocytes and neurons in lower glucose condition.

In the third series of experiments, we tested if the uptake of exogenous lactate via MCT1 and MCT2 can support neural network activity when the availability of glucose is strongly reduced (Figure 4). Low glucose (1 mM) strongly reduced gamma oscillations by 86% compared with normal glucose (5 mM) (gamma oscillations in 1 out of 7 slices versus 11 out of 11 slices, *p* = 0.0004, Fisher's exact test), whereas additional application of high lactate (8 mM) (Figure 4a) was associated with partial recovery (Figure 4c–f). The fraction of slices showing gamma oscillations in the presence of low glucose plus high lactate (Figure 4f) was reduced by 46% compared with normal glucose (Figure 2f) (gamma oscillations in 7 out of 13 slices versus 11 out of 11 slices, *p* = 0.016, Fisher's exact test). This finding was in line with previous reports demonstrating that supplemental lactate is less efficient in fueling network activities featuring high energy demand (Cox and Bachelard 1988; Galow et al. 2014; Hollnagel et al. 2020). The lower efficiency of lactate might reflect a portion of obligatory ATP synthesis in neurons by aerobic glycolysis, for example, in presynaptic terminals (varicosities) lacking mitochondria, and/or limitations in lactate flux through MCTs and LDH (Dienel 2019a; Galow et al. 2014; Hollnagel et al. 2020; Kann 2024; Shepherd and Harris 1998). Notably, the application of AR‐C155858 fully suppressed gamma oscillations in the presence of low glucose plus high lactate (Figure 4b–f), indicating the effective blockade of cellular lactate uptake by this compound in our experimental conditions.

Collectively, these data suggest that lactate is produced in cultured slices, but lactate transport via MCT1 and MCT2 is not required to sustain gamma oscillations, even when supply with glucose only is lowered. Lactate transport becomes important, however, when the glucose concentration is too low and lactate is available as supplemental fuel.

### 3.3 MCT1/2 Inhibition During Sharp Wave‐Ripples in Acute Slices

We also tested the effects of AR‐C155858 during sharp wave‐ripples in acute hippocampal slices. In this preparation, sharp wave‐ripples are spontaneously generated in the hippocampal CA3 network (Fischer et al. 2014; Schlingloff et al. 2014; Schneider et al. 2019), and they are quite stable for > 1 h in interface recording chambers (Hollnagel et al. 2020; Pollali and Draguhn 2025).

Sharp wave‐ripples reliably occurred in the presence of glucose (5 mM). These events reflect recurrent sharp waves with an incidence of about 3/s that are superimposed by fast oscillations at around 250 Hz (“ripples”) (Figure 5a,b). Notably, the subsequent application of AR‐C155858 (Figure 5c) in the same slices did neither affect the properties of sharp waves (Figure 5d,e) nor the fast “ripples” component (Figure 5f).

These data suggest that lactate transport via MCT1 and MCT2 is also not required to sustain sharp wave–ripples in acute slices.

### 3.4 MCT2 Inhibition in Primary Hippocampal Neurons

Our experiments (Figure 1d) as well as previous reports showed that lactate is produced in cultured slices (Chausse et al. 2020; Gramsbergen et al. 2003) as well as in acute slices of the rodent hippocampus (Andersen et al. 2021; Hall et al. 2012). It is still debated, however, whether this lactate is produced and released by astrocytes (Sotelo‐Hitschfeld et al. 2015; Suzuki et al. 2011; Walls et al. 2009; Zuend et al. 2020), and/or by activated neurons (Díaz‐García et al. 2017; Dienel 2019a; Gandhi et al. 2009; Mangia et al. 2009; Waagepetersen et al. 2000).

We therefore investigated the lactate dynamics in primary hippocampal neurons co‐cultured with astrocytes by combining the genetically encoded FRET reporter Laconic under control of the human syn promoter for specific expression in neurons (San Martín et al. 2013) and live‐cell fluorescence imaging. The experiments were performed in the presence of glucose (2 mM) and normal air that results in high oxygen levels in cell cultures (Section 4). The Laconic fluorescence signal was recorded in neuronal cell bodies (somas) (Figure 1e).

**FIGURE 6:**
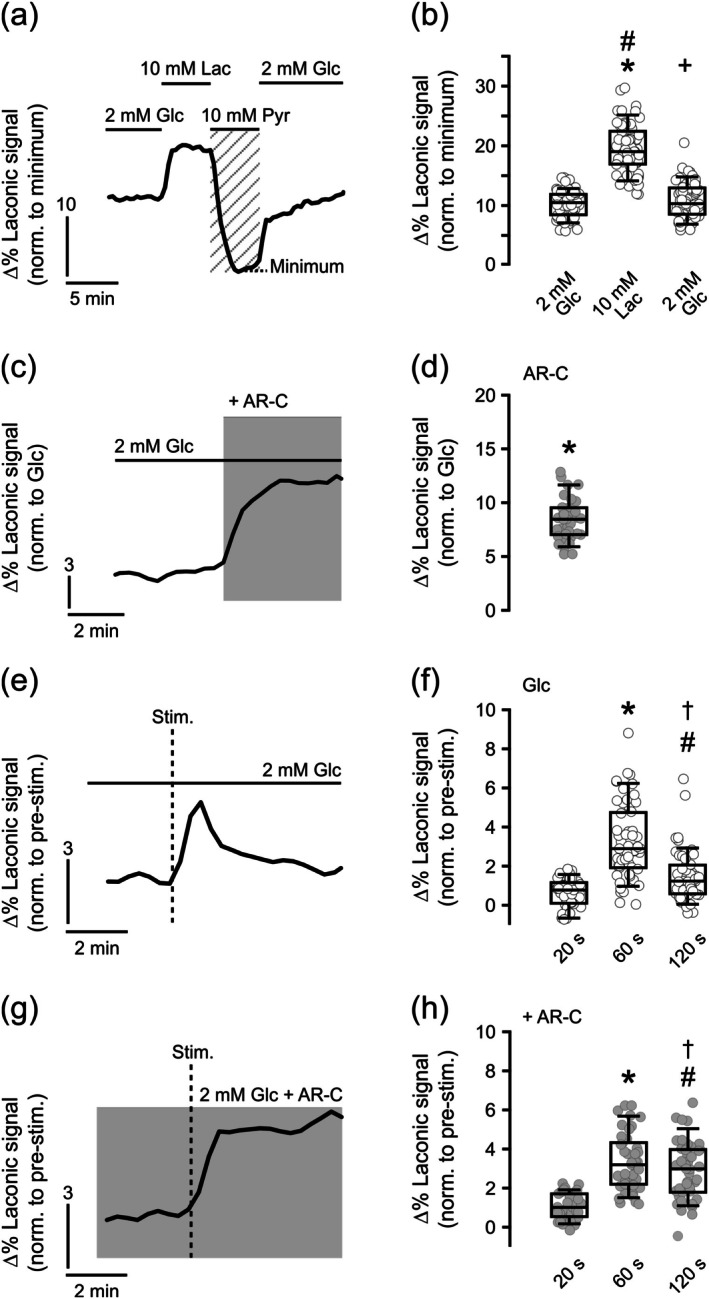
Lactate production and MCT2 inhibition in primary hippocampal neurons. (a) Representative trace of a two‐point Laconic calibration measured in the cell body of an individual neuron (Figure 1e). For Laconic calibration, neurons maintained in 2 mM glucose (Glc) were superfused with 10 mM lactate (Lac) followed by 10 mM pyruvate (Pyr) to obtain maximum and minimum of Laconic fluorescence emission intensity (Δ% Laconic signal), respectively. The minimum was achieved by transacceleration (Section 4). (b) Summary of the steady‐state level of lactate in neurons superfused with glucose before and after 10 mM lactate, normalized to the minimum. For *n*/*N* (cells/coverslips): 65/7. Data were compared with repeated measures ANOVA (Friedman test) followed by Durbin‐Conover pairwise comparisons test. *p < 0.001 versus Glc before, #*p* < 0.001 versus Glc after, *+p* = 0.023 Glc after versus before. (c) Representative trace of the transport‐stop protocol using the MCT1/2 inhibitor AR‐C155858 (AR‐C) (2 μM) in 2 mM glucose. (d) Summary of changes in Laconic signal (Δ%) during MCT1/2 blockade. For *n*/*N* (cells/coverslips): 35/5. Data were compared with paired *t*‐test. *p < 0.001 versus Glc only. (e) Representative trace of the Laconic signal during electrical field stimulation (Stim.) (10 Hz, 10 s) in 2 mM glucose. (f) Summary of changes in Laconic signal (Δ%) 20 s, 60 s, and 120 s after the onset of stimulation. For *n*/*N* (cells/coverslips): 65/4. Data were compared with repeated measures ANOVA followed by Durbin‐Conover test. *p < 0.001 versus 20 s, #*p* < 0.001 versus 20 s, †*p* < 0.001 versus 60 s. (g) Representative trace of the Laconic signal during electrical field stimulation (Stim.) (10 Hz, 10 s) in 2 mM glucose and AR‐C155858 (2 μM). (h) Summary of changes in Laconic signal (Δ%) 20 s, 60 s, and 120 s after the onset of stimulation. For *n*/*N* (cells/coverslips): 43/3. Data were compared with repeated measures ANOVA followed by Durbin‐Conover test. *p < 0.001 versus 20 s, #*p* < 0.001 versus 20 s, †*p* < 0.001 versus 60 s. Box plots indicate the middle 50% of the data and the median, whiskers show the 5% to 95% range.

In the first series of experiments, we sequentially switched from glucose (2 mM) to lactate (10 mM) to pyruvate (10 mM) for 4–5 min each to obtain maximal and minimal fluorescence emission intensity of the Laconic sensor (Figure 6a,b). Maximal fluorescence intensity was detected in lactate and reflected Laconic sensor saturation (San Martín et al. 2013). Minimal fluorescence intensity was detected with pyruvate and reflected neuronal lactate depletion by transacceleration (Section 4) (Baeza‐Lehnert et al. 2020; Mächler et al. 2016). The dynamic range of the Laconic signal was about 20Δ%, in agreement with the Δ%_Max_ of the purified Laconic protein (San Martín et al. 2013). This experiment revealed a substantial basal lactate level in cultured neurons in the presence of glucose only, even at high oxygenation.

In the second series of experiments, we applied AR‐C155858 in the presence of glucose (2 mM) as the only energy substrate. The blocker evoked a rapid increase in Laconic fluorescence intensity from baseline that saturated after about 2 min (Figure 6c,d). This finding likely reflects the imbalance between the flux of glucose through glycolysis and the flux of pyruvate oxidation in mitochondria. Furthermore, it shows both production and continuous release of lactate from cultured neurons during spontaneous activity (“resting state”). This finding was in line with previous reports and might partially reflect facilitated lactate release to the large extracellular medium volume (extracellular space) artifactually enhancing glycolysis in vitro (Dienel 2019a; Jekabsons et al. 2017; Waagepetersen et al. 2000).

In the third series of experiments, we induced neuronal activation by electrical field stimulation (10 Hz, 10 s) that enhances action potential firing and synaptic transmission, including metabolic adaptation. The stimulation induced a transient increase in Laconic fluorescence intensity by about 2Δ% in the presence of glucose (2 mM) that declined within 60 s (Figure 6e,f). This finding likely reflects activity‐dependent neurometabolic coupling and glucose metabolism. Likewise, electric stimulus‐induced neuronal activation with glucose (2 mM) in the presence of AR‐C155858 evoked the rapid saturation of Laconic fluorescence intensity (Figure 6g,h).

Collectively, these data suggest that lactate is produced in primary hippocampal neurons during spontaneous activity and stimulus‐induced activation and released via the neuronal MCT2.

## 4 Discussion

### 4.1 Exploring the Energetics Underlying Neural Oscillations in Hippocampal Slices

The contribution of lactate in fueling synaptic transmission during fast neural network oscillations associated with complex cortex function such as perception, memory formation, and motor activity is still debated (Colgin 2016; Dienel 2019a; Kann 2024; Magistretti and Allaman 2018; Nortley and Attwell 2017). Here, we explored lactate production as well as lactate transport via glial MCT1 and neuronal MCT2 in synchronized synaptic transmission underlying two different types of fast hippocampal network oscillations.

We investigated (i) gamma oscillations (around 40 Hz) in cultured slices, (ii) sharp wave‐ripples (with “ripples” around 250 Hz) in acute slices, and (iii) stimulus‐induced activation in primary neurons co‐cultured with astrocytes. The used cultured slices naturally mature during cultivation and corresponded to an about 3 weeks old hippocampus in vivo (Bahr et al. 1995; De Simoni et al. 2003; Tsintsadze et al. 2015). The acute slices were prepared from 4 to 6 weeks old mice. Our experimental approach thus comprised different types of neuronal activity, species (rat, mouse), preparations (ex vivo, in vitro), and developmental stages (juvenile, young adult) to draw more general conclusions. Moreover, all slice experiments were performed in the absence of anesthetics that can significantly interfere with synaptic transmission and energy metabolism (Dienel 2019a; Kann 2024).

Gamma oscillations occur in various cortex regions in awake mammals, including humans, during visual perception, motor activity, and memory formation, for example (Cheyne et al. 2008; Colgin 2016; Gray et al. 1989; Stauch et al. 2022; van Vugt et al. 2010). They support the timing of action potentials and synaptic plasticity. Sharp wave‐ripples arise in the hippocampus during resting periods and slow‐wave sleep (Buzsáki 2015; Ramirez‐Villegas et al. 2015). They are thought to transfer compressed hippocampal information to distributed neocortical circuits in memory consolidation (Buzsáki 2015; Colgin 2016). Both types of network oscillations require synchronized mutual synaptic transmission between excitatory glutamatergic pyramidal cells and different types of inhibitory GABAergic interneurons such as cholecystokinin‐positive, regular‐spiking basket cells and parvalbumin‐positive, fast‐spiking basket cells (Gulyás et al. 2010; Schlingloff et al. 2014). This mutual communication is reflected by excitatory and inhibitory postsynaptic currents underlying gamma oscillations (Gulyás et al. 2010; Traub et al. 1996) and sharp wave‐ripples (Schieferstein et al. 2024; Schlingloff et al. 2014). Gamma oscillations feature higher energy demand compared with sharp wave‐ripples (Hollnagel et al. 2020; Schneider et al. 2019), and they are exquisitely sensitive to metabolic and oxidative stress (Elzoheiry et al. 2020; Kann 2016; Kann et al. 2011; Papageorgiou et al. 2016).

In slice preparations, there are steep concentration gradients of glucose and oxygen from the slice surface to the slice core (Huchzermeyer et al. 2013; Lourenço et al. 2019; Schneider et al. 2019). In cultured slices supplied with 20% O_2_ fraction (similar to normal air), for example, the pO_2_ at the slice surface was around 140 mmHg (high oxygenation). In the slice core, the pO_2_ dropped to around 47 and 18 mmHg during spontaneous activity and gamma oscillations, respectively (Huchzermeyer et al. 2013). The lower concentrations of glucose and oxygen in the slice core reflect the inherent absence of blood circulation as well as diffusion, transport, and activity‐dependent consumption of these molecules (Kann 2024). To avoid metabolic disturbances of gamma oscillations and sharp wave‐ripples, we performed most of the slice experiments in the presence of 5 mM glucose and 95% O_2_ fraction (Galow et al. 2014; Huchzermeyer et al. 2013; Schneider et al. 2019). The resulting high tissue oxygenation also warranted that endogenously produced lactate could be oxidized in neuronal and glial mitochondria (Dienel 2019a; Kann 2024).

Similarly, we performed neuron‐astrocyte culture experiments in the presence of 2 mM glucose and normal air that had direct access to the cell monolayer (high cellular oxygenation), without any relevant concentration gradient compared with slices. Primary hippocampal neurons were cultured in the presence of astrocytes, which promote structural, functional, and metabolic differentiation (Mamczur et al. 2015; Perez‐Alvarez et al. 2014).

### 4.2 Activity‐Dependent Lactate Metabolism in Hippocampal Slices and Cultured Neurons

In cultured slices exhibiting gamma oscillations, we measured a tissue lactate concentration of about 1.5 μmol/g wet weight (Figure 1). These data suggest endogenous lactate production by neurons (see below) and glial cells, even at high tissue oxygenation. Fast gentle removal of Biopore membrane inserts from the recording chamber, including rapid freezing in normal air (Section 2) likely minimized anaerobic lactate production during tissue collection (Dienel 2021). Endogenous lactate production was also reported in slice cultures supplied with normal air and 5 mM glucose via perfusion (Gramsbergen et al. 2003) or 4 mM glucose via static culture medium (Chausse et al. 2024). In both studies, the neuronal network activity state was not assessed, however. Tissue lactate concentrations of about 1.7 μmol/g wet weight were also determined in cerebral cortex tissue after sensory stimulation of conscious rats (Dienel et al. 2002). Moreover, small but sustained increases in lactate were observed during visual stimulation and motor activation using functional magnetic resonance spectroscopy (fMRS) in healthy human volunteers (Bednařík et al. 2018; Mangia et al. 2009; Schaller et al. 2014). Collectively, the findings of lactate production at normal and high tissue oxygenation support the concept of aerobic glycolysis in the brain (Barros et al. 2021; Cunnane et al. 2020; Dienel 2019a; Fox et al. 1988).

Using live‐cell fluorescence imaging with the Laconic sensor in neuron‐astrocyte cultures, we found that lactate is produced in primary hippocampal neurons supplied with 2 mM glucose only, even at high cellular oxygenation (Figure 6). We identified this lactate production by transiently applying exogenous pyruvate at high concentration (10 mM). High pyruvate widely depletes intracellular lactate levels by so‐called transacceleration, where the presence of extracellular monocarboxylates (in this case pyruvate) stimulates substrate efflux via MCTs (in this case lactate efflux through MCT2) (Baeza‐Lehnert et al. 2020; Brown and Brooks 1994; Mächler et al. 2016; San Martín et al. 2013). The endogenous neuronal lactate production in glucose only (2 mM) likely results from glucose metabolism required for ATP production to fuel neuronal housekeeping and ion pumps, including the vesicle cycle because cultured hippocampal neurons feature spontaneous activity, that is, action potentials and synaptic transmission, in standard recording solutions. This functional “resting state” in culture is quite heterogeneous, however, with only a minority of cells firing action potentials at a high rate (Baeza‐Lehnert et al. 2019; Ivenshitz and Segal 2010; Panas et al. 2015; Pulido and Ryan 2021). When supplied with 2 mM glucose plus 1 mM lactate, primary hippocampal neurons become either “producers” or “consumers” of lactate (Baeza‐Lehnert et al. 2019), which likely depends on neuronal subtypes as well as developmental and activity states (Kann 2024). During electrical stimulation (10 Hz, 10 s), we found transient rises in the neuronal lactate concentration (Figure 6), similar to reports from granule neurons in acute hippocampal slices (Díaz‐García et al. 2017). This finding suggests activity‐dependent neurometabolic coupling and glucose metabolism as well as the transient surplus in neuronal pyruvate (and thus lactate) production by glycolysis compared with pyruvate oxidation in mitochondria.

Application of the specific high‐affinity blocker AR‐C155858 (Baeza‐Lehnert et al. 2019; Díaz‐García et al. 2017; Ovens, Davies, et al. 2010; Ovens, Manoharan, et al. 2010) finally revealed that primary hippocampal neurons co‐cultured with astrocytes release lactate via MCT2 during spontaneous activity as well as stimulus‐induced activation in the presence of glucose only (Figure 6). Neuronal lactate transients during MCT1/2 blockade might also argue against lactate shuttling from astrocytes to neurons during membrane depolarization (Sotelo‐Hitschfeld et al. 2015). These findings support the concept of neuronal lactate release to the extracellular space and the blood circulation at physical rest, which may involve the gap junction‐coupled astrocytic syncytium (Díaz‐García et al. 2017; Gandhi et al. 2009; Gramsbergen et al. 2003; Rothman et al. 2022).

Although lactate is produced in hippocampal tissue, including neurons (Figures 1 and 6), the blocker AR‐C155858 did neither alter gamma oscillations in cultured slices (Figure 2) nor sharp wave‐ripples in acute slices (Figure 5) when fueled with glucose. AR‐C155858 did also not alter gamma oscillations in the presence of lower glucose that likely associated with glycogen degradation and concomitant lactate production (Figure 3 and control experiment with DAB) (Cater et al. 2001; Galow et al. 2014; Gramsbergen et al. 2003; Oe et al. 2016). Interpretation of these data is somewhat limited, however, because we did not directly quantify lactate production in astrocytes. In the presence of high glucose and oxygen, pharmacological blockade of glycogenolysis did not affect gamma oscillations in cultured slices (Galow et al. 2014). Collectively, these findings suggest that transport as well as exchange of endogenous lactate via glial MCT1 and neuronal MCT2 is not required to sustain synchronized synaptic transmission when glucose is present. This might also support the view that astrocytic glycogen has an indirect role in preserving extracellular glucose for neuronal utilization, rather than directly providing lactate to neurons, at least in pathophysiological conditions with moderate metabolic and/or oxidative stress (Dienel et al. 2023, 2025; DiNuzzo et al. 2010; Rothman et al. 2022). In addition, glycogen utilization in astrocytes has been discussed to sequester inorganic phosphate (Pi), thereby increasing energy yield at sites of rapid ATP hydrolysis (Swanson 2020).

The metabolic interactions between neurons and astrocytes may significantly change during severe forms of glucose deprivation, however. Gamma oscillations were fully suppressed by MCT1/2 blockade when cultured slices mainly received exogenous lactate (Figure 4). This shows that neurons become “consumers” of lactate at increasing extracellular lactate/glucose ratios suggestive of metabolic flexibility (Antal et al. 2025; Baeza‐Lehnert et al. 2019; Chausse et al. 2024; Galow et al. 2014; Hollnagel et al. 2020; Matsui et al. 2017; Solís‐Maldonado et al. 2018). During full glucose deprivation (in the presence of oxygen), the breakdown of glycogen significantly delayed the suppression of gamma oscillations in cultured slices (Galow et al. 2014), which may also reflect glial lactate supply to neurons in pathological conditions (Cater et al. 2001; Dringen et al. 1993; Gramsbergen et al. 2003; Mangia et al. 2009).

## 5 Conclusions

Our results show that lactate is produced in cortical tissue, including neurons fueled by glucose only. Moreover, lactate transport and lactate exchange via glial MCT1 and neuronal MCT2 are not required for synchronized synaptic transmission underlying fast neural network oscillations. These findings may contribute to the ongoing debate about aerobic glycolysis, astrocyte‐neuron shuttling of lactate derived from glucose and glycogen, excitatory and inhibitory synaptic transmission, and fast neural network oscillations that underlie higher brain functions.

## Author Contributions

**Lennart Söder:** conceptualization, investigation, formal analysis, visualization, writing – original draft, writing – review and editing, data curation. **Felipe Baeza‐Lehnert:** conceptualization, investigation, formal analysis, visualization, writing – review and editing, funding acquisition, data curation. **Babak Khodaie:** investigation, formal analysis, visualization, writing – review and editing, data curation. **Amr Elgez:** investigation, formal analysis, writing – review and editing. **Lena Noack:** investigation, formal analysis, writing – review and editing. **Andrea Lewen:** investigation, formal analysis, visualization, writing – review and editing, project administration. **Stefan Hallermann:** formal analysis, writing – review and editing, funding acquisition. **Gernot Poschet:** investigation, formal analysis, writing – review and editing. **Karin Borges:** formal analysis, writing – review and editing. **Oliver Kann:** conceptualization, formal analysis, visualization, supervision, project administration, writing – original draft, writing – review and editing.

## Conflicts of Interest

The authors declare no conflicts of interest.

## Peer Review

The peer review history for this article is available at https://www.webofscience.com/api/gateway/wos/peer‐review/10.1111/jnc.70251.

## Supporting information


**Table S1:** jnc70251‐sup‐0001‐TableS1.pdf.

## Data Availability

The data that support the findings of this study are available from the corresponding author upon reasonable request.
