# Supplementary material for: Lactate Transport via Glial MCT1 and Neuronal MCT2 Is Not Required for Synchronized Synaptic Transmission in Hippocampal Slices Supplied With Glucose
Source: J Neurochem. 2025 Oct 6;169(10):e70251. doi: 10.1111/jnc.70251 (PMC12498266; doi:10.1111/jnc.70251)
Supplement: Supplementary file 1 — Table S1: jnc70251‐sup‐0001‐TableS1.pdf. [file JNC-169-0-s001.pdf]

Lactate transport via glial MCT1 and neuronal MCT2 is not required for synchronized synaptic transmission in hippocampal slices supplied with glucose

Lennart Söder[1], Felipe Baeza-Lehnert[2], Babak Khodaie[1], Amr Elgez[1], Lena Noack[1], Andrea Lewen[1], Stefan Hallermann[2], Gernot Poschet[3], Karin Borges[4] and Oliver Kann[1,5]

[1]Institute of Physiology and Pathophysiology, Heidelberg University, Heidelberg, Germany, [2]Carl-Ludwig-Institute of Physiology, Faculty of Medicine, Leipzig University, Leipzig, Germany, [3]Metabolomics Core Technology Platform, Centre for Organismal Studies, Heidelberg University, Heidelberg, Germany, [4]School of Biomedical Sciences, Faculty of Health, Medicine and Behavioural Sciences, The University of Queensland, Brisbane, Australia, [5]Interdisciplinary Center for Neurosciences (IZN), Heidelberg University, Heidelberg, Germany

Supplementary Material containing Supplemental Table S1.

Lactate transport via glial MCT1 and neuronal MCT2 is not required for synchronized synaptic transmission in hippocampal slices supplied with glucose

Lennart Söder, Felipe Baeza-Lehnert, Babak Khodaie, Amr Elgez, Lena Noack, Andrea Lewen, Stefan Hallermann, Gernot Poschet, Karin Borges and Oliver Kann

Supplemental Table S1.

Figures	Parameter	Groups compared ([A] vs [B])	Statistical test	P value	Degrees of freedom	Test details
1d	Lac (µmol/g wet weight)	[5 mM Glc] vs [1 mM Glc + 8 mM Lac]	Unpaired t-test, two-tailed	0.0016	7	t = 5.003
2c	Frequency (Hz)	[5 mM Glc] vs [5 mM Glc + AR-C]	Unpaired t-test, two-tailed	0.3386	19	t = 0.9816
2d	Power (mV^2/Hz)	[5 mM Glc] vs [5 mM Glc + AR-C]	Mann-Whitney's test	0.5573		U = 46
2e	FWHM (Hz)	[5 mM Glc] vs [5 mM Glc + AR-C]	Unpaired t-test, two-tailed	0.5668	19	t = 0.5829
2f	GAM vs NO GAM	[5 mM Glc] vs [5 mM Glc + AR-C]	Fisher's exact test	>0.9999		
3c	Frequency (Hz)	[2.5 mM Glc] vs [2.5 mM Glc + AR-C]	Unpaired t-test, two-tailed	0.3780	22	t = 0.8998
3d	Power (mV^2/Hz)	[2.5 mM Glc] vs [2.5 mM Glc + AR-C]	Unpaired t-test, two-tailed	0.2302	22	t = 1.234
3e	FWHM (Hz)	[2.5 mM Glc] vs [2.5 mM Glc + AR-C]	Unpaired t-test, two-tailed	0.7452	22	t = 0.3291
3f	GAM vs NO GAM	[2.5 mM Glc] vs [2.5 mM Glc + AR-C]	Fisher's exact test	>0.9999		
4f	GAM vs NO GAM	[1 mM Glc + 8 mM Lac] vs [1 mM Glc + 8 mM Lac + AR-C]	Fisher's exact test	0.0059		

Lactate transport via glial MCT1 and neuronal MCT2 is not required for synchronized synaptic transmission in hippocampal slices supplied with glucose

Lennart Söder, Felipe Baeza-Lehnert, Babak Khodaie, Amr Elgez, Lena Noack, Andrea Lewen, Stefan Hallermann, Gernot Poschet, Karin Borges and Oliver Kann

Figures	Parameter	Groups compared ([A] vs [B])	Statistical test	P value	Degrees of freedom	Test details
5d	SPW amplitude (mV)	[5 mM Glc] vs [5 mM Glc + AR-C]	Wilcoxon rank test	0.4961		W = 13
5e	SPW incidence/s	[5 mM Glc] vs [5 mM Glc + AR-C]	Paired t-test, two-tailed	0.0859	8	t = 1.958
5f	Ripple frequency (Hz)	[5 mM Glc] vs [5 mM Glc + AR-C]	Paired t-test, two-tailed	0.1164	8	t = 1.760
6b	Δ% Laconic signal (norm. to minimum)	[2 mM Glc (initial)] vs [10 mM Lac] vs [2 mM Glc (final)]	Repeated measure non-parametric ANOVA (Friedman test) followed by Durbin-Conover pairwise comparison test (see below)	<0.001	2	X^2=98.8
6b	Δ% Laconic signal (norm. to minimum)	[2 mM Glc (initial)] vs [10 mM Lac]	Pairwise comparisons (Durbin-Conover)	<0.001		z = 18.48
6b	Δ% Laconic signal (norm. to minimum)	[2 mM Glc (initial)] vs [2 mM Glc (final)]	Pairwise comparisons (Durbin-Conover)	0.023		z = 2.31
6b	Δ% Laconic signal (norm. to minimum)	[10 mM Lac] vs [2 mM Glc (final)]	Pairwise comparisons (Durbin-Conover)	<0.001		z = 16.17
6d	Δ% Laconic signal (norm. to Glc)	[2 mM Glc] vs [2 mM Glc + AR-C]	Paired t-test, two-tailed	<0.001	35	z = -26.3

Lactate transport via glial MCT1 and neuronal MCT2 is not required for synchronized synaptic transmission in hippocampal slices supplied with glucose

Lennart Söder, Felipe Baeza-Lehnert, Babak Khodaie, Amr Elgez, Lena Noack, Andrea Lewen, Stefan Hallermann, Gernot Poschet, Karin Borges and Oliver Kann

Figures	Parameter	Groups compared ([A] vs [B])	Statistical test	P value	Degrees of freedom	Test details
6f	Δ% Laconic signal (norm. to pre-stim.)	[20 s] vs [60 s] vs [120 s]	Repeated measure non-parametric ANOVA (Friedman test) followed by Durbin-Conover pairwise comparison test (see below)	<0.001	2	X^2=82.8
6f	Δ% Laconic signal (norm. to pre-stim.) 20 s vs 60 s	[20 s] vs [60 s]	Pairwise comparisons (Durbin-Conover)	<0.001		z = 17.21
6f	Δ% Laconic signal (norm. to pre-stim.) 20 s vs 120 s	[20 s] vs [120 s]	Pairwise comparisons (Durbin-Conover)	<0.001		z = 7.80
6f	Δ% Laconic signal (norm. to pre-stim.)	[60 s] vs [120 s]	Pairwise comparisons (Durbin-Conover)	<0.001		z = 9.40
6h	Δ% Laconic signal (norm. to pre-stim.)	[20 s] vs [60 s] vs [120 s]	Repeated measure non-parametric ANOVA (Friedman test) followed by Durbin-Conover pairwise comparison test (see below)	<0.001	2	X^2=65.9
6h	Δ% Laconic signal (norm. to pre-stim.)	[20 s] vs [60 s]	Pairwise comparisons (Durbin-Conover)	<0.001		z = 16.32
6h	Δ% Laconic signal (norm. to pre-stim.)	[20 s] vs [120 s]	Pairwise comparisons (Durbin-Conover)	<0.001		z = 10.80
6h	Δ% Laconic signal (norm. to pre-stim.)	[60 s] vs [120 s]	Pairwise comparisons (Durbin-Conover)	<0.001		z = 5.51

Lactate transport via glial MCT1 and neuronal MCT2 is not required for synchronized synaptic transmission in hippocampal slices supplied with glucose

Lennart Söder, Felipe Baeza-Lehnert, Babak Khodaie, Amr Elgez, Lena Noack, Andrea Lewen, Stefan Hallermann, Gernot Poschet, Karin Borges and Oliver Kann

**Section in main text**	**Parameter**	**Groups compared ([A] vs [B])**	**Mean ± standard deviation, n/N refers to slices/animals**<br><br>**Statistical test**	**P value**	**Degrees of freedom**	**Test details**
3.2	Frequency (Hz)	[2.5 mM Glc] vs [2.5 mM Glc + DAB]	41.10 ± 3.77 (n/N = 12/4) versus 40.38 ± 4.06 (n/N = 6/4)<br><br>Unpaired t-test, two-tailed	0.7173	16	t = 0.3685
3.2	Power (mV^2/Hz)	[2.5 mM Glc] vs [2.5 mM Glc + DAB]	0.001040 ± 0.0007 (n/N = 12/4) versus 0.000394 ± 0.0001 (n/N = 6/4)<br><br>Unpaired t-test, two-tailed	0.0446	16	t = 2.179
3.2	FWHM (Hz)	[2.5 mM Glc] vs [2.5 mM Glc + DAB]	22.90 ± 9.68 (n/N = 12/4) versus 34.21 ± 5.95 (n/N = 6/4)<br><br>Unpaired t-test, two-tailed	0.0191	16	t = 2.605
3.2	GAM vs NO GAM	[5 mM Glc] vs [1 mM Glc]	Fisher's exact test	0.0004		
3.2	GAM vs NO GAM	[1 mM Glc + 8 mM Lac] vs [5 mM Glc]	Fisher's exact test	0.0162		
